# Transcriptome Analysis of Zygotic Induction During Conjugative Transfer of Plasmid RP4

**DOI:** 10.3389/fmicb.2020.01125

**Published:** 2020-06-18

**Authors:** Masatoshi Miyakoshi, Yoshiyuki Ohtsubo, Yuji Nagata, Masataka Tsuda

**Affiliations:** ^1^Department of Molecular and Chemical Life Sciences, Graduate School of Life Sciences, Tohoku University, Sendai, Japan; ^2^Division of Biomedical Science, Faculty of Medicine, University of Tsukuba, Tsukuba, Japan

**Keywords:** transcriptome, conjugative transfer, relaxosome, RP4, zygotic induction

## Abstract

Conjugative transfer of bacterial plasmid is one of the major mechanisms of horizontal gene transfer, which is mediated by direct contact between donor and recipient cells. Gene expression of a conjugative plasmid is tightly regulated mostly by plasmid-encoded transcriptional regulators, but it remains obscure how differently plasmid genes are expressed in each cell during the conjugation event. Here, we report a comprehensive analysis of gene expression during conjugative transfer of plasmid RP4, which is transferred between isogenic strains of *Pseudomonas putida* KT2440 at very high frequency. To discriminate the expression changes in the donor and recipient cells, we took advantage of conjugation in the presence of rifampicin (Rif). Within 10 min of mating, we successfully detected transient transcription of plasmid genes in the resultant transconjugant cells. This phenomenon known as zygotic induction is likely attributed to derepression of multiple RP4-encoded repressors. Interestingly, we also observed that the *traJIH* operon encoding relaxase and its auxiliary proteins were upregulated specifically in the donor cells. Identification of the 5′ end of the zygotically induced *traJ* mRNA confirmed that the transcription start site of *traJ* was located 24-nt upstream of the nick site in the origin of transfer (*oriT*) as previously reported. Since the *traJ* promoter is encoded on the region to be transferred first, the relaxase may be expressed in the donor cell after regeneration of the *oriT-*flanking region, which in itself is likely to displace the autogenous repressors around *oriT*. This study provides new insights into the regulation of plasmid transfer processes.

## Introduction

Plasmids are extra-chromosomal genetic elements that replicate autonomously by plasmid-encoded elements in cooperation with the host cell chromosome and are vertically inherited by cell division through active partitioning, multimer resolution, and post-segregational killing mechanisms. They can also be propagated horizontally by conjugative transfer through direct contact between donor and recipient cells (Thomas and Nielsen, 2005). In Gram-negative bacteria, plasmids are replicated commonly by the theta replication system during vegetative growth and also by the rolling-circle replication (RCR) system during conjugative transfer (Willetts and Wilkins, 1984; Waters and Guiney, 1993; Lanka and Wilkins, 1995; Llosa et al., 2002; de la Cruz et al., 2010). The origins of the two modes of plasmid replication are designated as *oriV* and *oriT*, respectively.

Self-transmissible plasmid is equipped with a conjugative transfer system mainly composed of a DNA processing machinery for transfer and replication (Dtr) and a type IV secretion system (T4SS) for mating pair formation (Mpf), the latter of which is embedded in membranes of a donor cell and penetrates into a recipient cell (Lawley et al., 2004; Cabezón et al., 2015; Waksman, 2019). A conjugative plasmid prepares for transfer through an assembly of protein complexes called relaxosome at the origin of transfer (*oriT*) region and then cleavage of the double-stranded plasmid DNA by relaxase, a class of the HUH endonuclease superfamily (Chandler et al., 2013). The relaxase specifically cleaves the nick site (*nic*) in *oriT* using a tyrosine residue in its catalytic transesterase domain, which covalently binds with the 5′-end phosphate of the transfer strand. The relaxase is recruited to the T4SS by a coupling protein, and both the relaxase and the single-stranded DNA (ssDNA) are transported unidirectionally with a 5′ to 3′ polarity from the donor to the recipient through the same T4SS conduit (Waksman, 2019). As the transfer strand is transported, both replacement and complementary strands are synthesized in the donor cell and recipient cell, respectively, yielding two copies of plasmid.

RP4 is the representative broad-host-range (BHR) self-transmissible plasmid belonging to IncP-1α incompatibility group whose sequence has been completed in 1994 (Pansegrau et al., 1994a). The life cycle of RP4 is largely independent of host factors, and its gene expression is regulated by a complex transcriptional circuit composed of autogenous transcription factors, namely, the global regulators KorA, KorB, KorC, and TrbA that bind at multiple sites on RP4 and the local DNA binding proteins such as TraJ and TraK (Thomas, 2000; Bingle and Thomas, 2001). The nature of negative regulation implies a transient expression of plasmid genes during conjugative transfer until the repressors reach a sufficient level in a new host cell (Thomas, 2000, 2006), but the actual range of induction has not been shown experimentally.

Transcriptomic analyses have revealed unprecedented aspects of plasmid biology, especially in crosstalks between plasmids and chromosomes (Nojiri, 2013; Vial and Hommais, 2020). We have been studying the impact of plasmid carriage on the regulatory network of host bacteria through plasmid-encoded elements (Miyakoshi et al., 2007; Shintani et al., 2010). Our studies have led to the discovery of a chromosomal ParA ATPase homolog that is encoded in a genomic island resided in *P. putida* strain KT2440 and inhibits the partitioning of a specific class of plasmid (Miyakoshi et al., 2007, 2012). A comparison of plasmid transcriptomes in several host bacteria have also shown that expression of plasmid genes is variable depending on the host genetic background (Miyakoshi et al., 2009; Shintani et al., 2011). By using the promiscuous plasmid RP4, we expected to detect drastic expression changes of plasmid genes in much broader range of host strains.

Originally proposed in Jacob and Wollman (1956), zygotic induction is the transient transcriptional activation that takes place in the early stages of conjugative transfer in recipient cells (Bagdasarian et al., 1992; Jones et al., 1992). This phenomenon is attributed to either stimulation of single-stranded promoters on the transfer strand, which are silenced by synthesis of the complementary strand (Masai and Arai, 1997; Bates et al., 1999) or derepression of plasmid genes in a shortage of plasmid-specified repressors. Taking advantage of RNA polymerase inhibitor rifampicin (Rif), the pioneering study in ColIb-P9 conjugative plasmid has detected zygotic induction of plasmid genes in recipient cells (Althorpe et al., 1999). These studies prompted us to comprehensively analyze temporal RNA products during conjugative transfer *in vivo*. To this end, we performed transcriptome analysis of the very efficient self-transmissible plasmid RP4 in the mating between rifampicin-resistant (Rif^R^) and -sensitive (Rif^s^) strains, and successfully showed not only the zygotic induction in *de novo* transconjugant cells but also the expression of relaxosome components in the donor cells during conjugative transfer.

## Materials and Methods

### Bacterial Strains

*Pseudomonas putida* strain KT2440 (ATCC47054) was used as the host of plasmid RP4. *P. putida* strain KT2442 is a spontaneous Rif^R^ mutant of KT2440, whose *rpoB* gene acquired an A to G mutation at the 1,553rd nucleotide (Gln518Arg).

Bacterial cells were aerobically grown in LB medium at 30°C. The following antibiotics were added to the media: kanamycin (Km, 50 μg/ml), rifampicin (Rif, 100 μg/ml). For plate cultures, the above media were solidified with 1.5% agar (wt/vol).

### RNA Extraction From Conjugating Cells

Each donor or recipient strain grown overnight was inoculated into a fresh 5-ml LB medium by 100-fold dilution. The cells grown to stationary phase (OD_600_ of 2.0) were harvested by centrifugation and resuspended into 500 μl of LB medium containing Rif. After mixing the donor and recipient cells in combinations of Rif^R^ and Rif^S^ isogenic strains, 100 μl of each mixture was immediately spotted on a sterile cellulose acetate membrane filter with 0.45-μm pore size (Advantec), which was placed onto LB agar plate containing Rif to allow the cells to conjugate at 30°C for 10 min. As controls, we spotted each donor or recipient cells separately on membrane filters, which were separately placed onto LB agar plate containing Rif and incubated at 30°C for 10 min.

The cells were released from the filter by vortexing in 1 ml of RNAprotect Bacteria Reagent (Qiagen), and the total RNA was extracted and purified using RNeasy Mini kit (Qiagen) according to the manufacturer’s instruction. The total RNA was treated by TURBO DNase (Ambion) at 37°C for 30 min and purified by RNeasy Cleanup (Qiagen). The RNA integrity was checked using 2100 Bioanalyzer (Agilent).

### Microarray Analysis

RNA samples were independently extracted in duplicate and subjected to NimbleGen oligonucleotide microarray (Roche Diagnostics). The custom microarray contains six pairs of 60-mer probes that hybridize with each of the 5,540 genes from the *P. putida* KT2440 chromosome and RP4 plasmid genomes. The cDNA synthesis, hybridization, and scanning were performed by Roche Diagnostics. The microarray data were analyzed by NANDEMO analysis software (Roche Diagnostics). The expression change during the 10-min conjugative transfer was calculated by the ratio of transcript levels in the RNA sample from the mixture of donor and recipient cells to the sum of the equal amount of two RNA samples, which are independently extracted from the donor and recipient cells.

### Reverse Transcription-Quantitative PCR (RT-qPCR)

Total RNA was extracted as described above. Reverse transcription was performed in 20-μl solution of 1 × First Strand Buffer containing 5 μg of total RNA, 125 ng of random primers (Invitrogen), 5 mM DTT, 0.5 mM dNTPs, 40 U of RNaseOUT (Invitrogen), and 200 U of SuperScript III (Invitrogen). After the RNA and random primers were denatured at 70°C for 10 min and annealed at 25°C for 10 min, the remaining reagents were added, and the mixture was incubated at 25°C for 10 min, 50°C for 60 min, and then held at 70°C for 15 min to inactivate the enzymes.

qPCR was performed using MiniOpticon real-time PCR system (BioRad). Each 20-μl reaction mixture contained 10 μl of 2 × SYBR Premix ExTaq (Takara), 200 nM concentrations of each specific primers and the appropriately diluted cDNA. The primer pairs used for qPCR were as follows: 16S-F (5′-ACACGGT CCAGACTCCTACG-3′) and 16S-R (5′-TACTGCCCTTCCTCC CAACT-3′), klcA-F (5′-TTCAAATCCCCTCCCCTATC-3′) and klcA-R (5′-CCATCCAGCCGAATACCAG-3′), and traJ-F (5′-CCTTCCAGACGAACGAAGAG-3′) and traJ-R (5′-GAC GTGCTCATAGTCCACGA-3′). The reaction condition was as follows: 95°C for 10 s for enzyme activation and 40 cycles of 95°C for 10 s and 60°C for 20 s. A melting curve analysis was performed to verify the amplification specificity. To quantify the transcription of each gene, the copy number was determined by generating a standard curve using a series of 10-fold dilutions (from 100 pM to 1 fM) of the target PCR product. For sample normalization, 16S rRNA was used as an internal standard. All of the reactions were performed in triplicate, and the data were normalized using the average of the internal standard.

### 5′RACE

5′RACE was performed according to the method described in Bensing et al. (1996). Briefly, 6 μg of total RNA was treated with 75 U of tobacco pyrophosphatase (TAP; Nippon Gene) at 37°C for 30 min in the presence of 20 U of RNaseOUT. The TAP-treated and -untreated RNA samples were mixed with the RNA oligonucleotide (5′-AUAUGCGCG AAUUCCUGUAGCUAGAAGAAA-3′) and ligated by 40 U of T4 RNA ligase (TAKARA Bio) at 16°C overnight. The ligated RNA samples were mixed with 1 pmol of gene-specific primer traJ-R2 (5′-TCTCTTCGATCTTCGCCAGC-3′) and reverse transcribed by 100 U of SuperScriptIII at 50°C for 60 min in the presence of 20 U of RNaseOUT. The cDNA fragment spanning the ligated RNA oligonucleotide and the 5′ end of *traJ* transcript was amplified by KOD-Plus high-fidelity DNA polymerase (TOYOBO) using primers Oligo-F1 (5′-TATGCGCGAATTCCTGTAGC-3′) and traJ-R. The amplified fragment was cloned into *Hin*cII-digested pBluescript II SK(-) vector (Stratagene), and the inserts from several clones were sequenced using M13 primers.

## Results and Discussion

### Experimental Design

**FIGURE 1:**
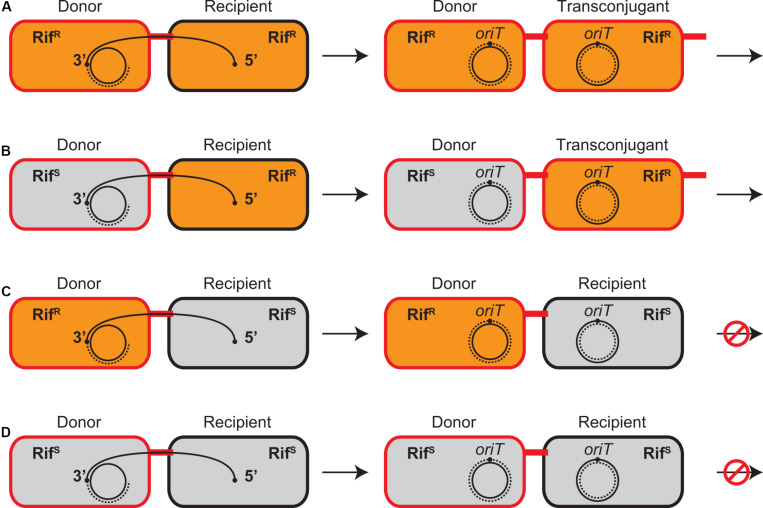
Conjugative transfer between combinations of Rif^R^ and Rif^S^ strains in the presence of Rif. Rif^R^ and Rif^S^ cells are shown as orange and gray bacilli, respectively. The donor and transconjugant cells proficient in plasmid transfer are indicated in red while the recipient cells are indicated in black. The transfer strand is represented as a solid curve and its *oriT* sites are indicated by black dots, which is transferred from the donor to the recipient in a direction from 5′ to 3′. The replacement and complementary strands are represented as broken lines. **(A)** Both donor and recipient are Rif^R^. **(B)** When recipient is Rif^R^, Rif does not prevent plasmid transfer from Rif^S^ donor and new transconjugant is established. **(C)** When recipient is Rif^S^, Rif prevents establishment of transconjugant but not transcription in Rif^R^ donor. **(D)** Both donor and recipient are Rif^S^. No transcription is initiated in the presence of Rif.

A conjugative plasmid is transferred from a donor cell to a recipient cell, the latter of which turns into an active transconjugant cell through zygotic induction of plasmid genes. The first conjugative transfer triggers a chain reaction of plasmid transfer from the *de novo* transconjugant cells to next recipient cells (Figure 1). The initial contact between the donor and recipient cells is stochastic, and the conjugation events cannot be synchronized. However, the very high transfer efficiency of RP4 between *P. putida* KT2440 (Bingle et al., 2003) allows maximizing the population of transconjugants in the mixture of cells. Given that the conjugative transfer of plasmids is conducted at the rate of 45 kb/min (as in the case of *E. coli* Hfr) (Lawley et al., 2004), the 60-kb RP4 plasmid can be transferred in 1.3 min and is sufficient to accomplish a single round of transfer within 10 min. In our conjugation experiment, ∼1 × 10^9^ cells of donor and recipient were mixed equally and allowed for mating on the filter membrane for 10 min. RP4 was transferred between isogenic KT2440 strains at the efficiency of >1 × 10^–1^ (CFU ratio of transconjugant/recipient), indicating that >10% of recipient cells acquired the plasmid in 10 min.

For simplicity, this study utilized the custom microarray containing both genomes of KT2440 chromosome and RP4 plasmid to analyze the conjugative transfer between the cells with the same genetic background. Assuming that there are no expression changes between transconjugant and donor cells, the transcript level of each RP4 gene is estimated to raise by twofold at the maximum when the transfer efficiency is 100% (the number of transconjugant cells is equal to that of donor cells). Cell growth can be ignored within the 10-min filter mating since the cells on the membrane filter are concentrated 10-fold from the stationary-phase cultures. Therefore, we set the threshold at fourfold change for upregulation of gene expression during conjugative transfer.

Although the donor and resultant transconjugant cells are genetically identical, transcription of plasmid genes in either cell is distinguishable using Rif and combinations of Rif^R^ and Rif^S^ isogenic strains, which are only different in *rpoB* (Figure 1). It has been known for F plasmid that the established Rif^S^ donors can transfer the plasmid into the recipients, while the Rif^S^ recipients fail to accomplish the plasmid transfer in the presence of Rif, indicating that *de novo* expression in the donor cells is dispensable for initiation of conjugative transfer (Wilkins and Hollom, 1974; Kingsman and Willetts, 1978). Similarly, our mating experiment in the 1:1 mixture of RP4 donor and recipient cells in the presence of Rif showed that the transfer efficiency of Rif^S^ donors was no greater than that of Rif^R^ donors and that neither donor strains were able to establish transconjugants in Rif^S^ recipients (data not shown).

### Zygotic Induction in *de novo* Transconjugant Cells

The mating between the Rif^S^ donor and Rif^R^ recipient strains generates the new Rif^R^ transconjugant strain, which is genetically identical to the Rif^R^ donor strain and continues conjugative transfer to the next recipient cells. Therefore, this combination is virtually identical to the mating between the Rif^R^ donor and Rif^R^ recipient strains irrespective of the initial donor’s genetic background (Figures 1A,B), although the number of resultant Rif^R^ donor cells is apparently smaller than the latter combination. We successfully detected the transcription upregulation of many plasmid genes in the *de novo* transconjugant cells in these two combinations (Table 1). The fold changes were generally smaller in the combination of Rif^S^ donor and Rif^R^ recipient (the second column) than that of Rif^R^ donor and Rif^R^ recipient (the first column). This result is likely to reflect the number of active transconjugant cells in the population.

**TABLE 1:** Expression changes of RP4 genes during conjugative transfer.

			Rif^R^ donor	Rif^S^ donor	Rif^R^ donor	Rif^S^ donor
						
Gene	Function	Direction^a^	Rif^R^ recipient	Rif^R^ recipient	Rif^S^ recipient	Rif^S^ recipient
*traK*	Relaxosome auxiliary protein	→	3.0	1.1	3.3	1.4
*traL*		→	3.2	1.4	1.9	1.2
*traM*		→	2.2	1.4	1.8	1.0
*kfrC*	Plasmid maintenance	←	3.0	2.4	1.6	1.2
*kfrB*	Plasmid maintenance	←	**7.3**	**5.4**	1.9	1.1
*kfrA*	Autoregulator protein	←	**16.5**	**5.7**	**4**.**9**	1.3
*korG*	Histone-like protein	←	**11.2**	**11.0**	1.0	1.2
*korF*	Histone-like protein	←	**14.8**	**14.5**	0.9	1.2
*korB*	Global transcription repressor	←	**15.8**	**14.9**	1.1	1.1
*incC*	Plasmid partitioning protein	←	**18.5**	**14.3**	1.3	1.1
*korA*	Global transcription repressor	←	**19.0**	**13.3**	1.5	1.0
*klaC*	Plasmid maintenance	←	**14.5**	**9.5**	0.9	1.3
*klaB*	Plasmid maintenance	←	**20.5**	**15.9**	1.1	1.2
*klaA*	Plasmid maintenance	←	**35.9**	**34.5**	1.0	1.3
*kleF*	Plasmid maintenance	←	**22.4**	**15.5**	1.1	1.2
*kleE*	Plasmid maintenance	←	**36.2**	**23.3**	0.9	1.2
*kleD*	Plasmid maintenance	←	**36.9**	**16.4**	1.1	1.4
*kleC*	Plasmid maintenance	←	**50.5**	**27.8**	0.9	1.5
*kleB*	Plasmid maintenance	←	**50.6**	**26.0**	0.7	1.7
*kleA*	Plasmid maintenance	←	**38.1**	**22.2**	0.8	1.5
*korC*	Global transcription repressor	←	0.8	1.2	0.7	1.5
*bla*	Beta-lactamase	←	1.3	1.3	1.2	1.2
*tnpR*	Tn*1* resolvase	←	2.8	2.0	1.7	1.4
*tnpA*	Tn*1* transposase	→	2.1	1.3	1.1	1.1
*klcA*	Antirestriction enzyme	←	**223.7**	**121.0**	0.9	1.3
*tetR*	*tetA* repressor	←	1.6	1.6	1.1	1.1
*tetA*	Tetracycline exporter	→	**7.0**	**4.7**	1.0	1.6
*upf16.5*		←	**7.4**	**7.0**	1.3	1.0
*trfA*	Replication initiator protein	←	**7.4**	**7.5**	1.4	1.1
*ssb*	ssDNA binding protein	←	**9.4**	**9.7**	1.3	1.2
*trbA*	Global transcription repressor	→	1.7	1.4	1.0	1.3
*trbB*	T4SS protein	→	**5.0**	**6.9**	1.5	1.3
*trbC*	P-type propilin	→	3.0	**4.9**	1.8	1.3
*trbD*	Plus assembly	→	2.6	3.0	1.5	1.3
*trbE*	T4SS ATPase	→	2.1	3.0	1.4	1.4
*trbF*	Plus assembly	→	1.5	2.3	1.5	1.6
*trbG*	T4SS protein	→	1.3	1.7	1.0	1.4
*trbH*	T4SS protein	→	1.0	1.5	0.8	1.5
*trbI*	T4SS protein	→	0.9	1.1	0.9	1.3
*trbJ*	T4SS protein	→	0.8	1.2	1.0	1.4
*trbK*	Entry exclusion protein	→	0.6	1.0	1.1	1.0
*trbL*	Mating pair formation protein	→	0.7	1.2	0.7	1.3
*trbM*	Mating pair formation protein	→	0.8	1.0	1.0	1.3
*trbN*	Mating pair formation protein	→	0.7	1.2	0.9	1.3
*trbO*		→	0.7	1.0	0.9	1.3
*trbP*	Putative pilus acetylase	→	0.6	1.1	0.7	1.6
*upf31.7*		→	0.7	1.0	0.8	1.4
*fiwA*	Fertility inhibition of IncW plasmids	→	0.6	1.1	0.8	1.2
*upf32.8*		→	1.2	1.4	0.8	1.2
*parA*	Site-specific recombinase	←	**10.5**	2.5	1.1	1.2
*parB*	Nuclease	←	**4.7**	2.9	1.6	0.9
*parC*		←	**10.6**	2.6	1.3	1.6
*parD*	Antitoxin protein	→	**6.8**	3.5	2.9	1.3
*parE*	Toxin protein	→	**6.7**	**4.7**	3.3	1.2
*istA*	IS*21* transposase	→	3.7	2.4	2.3	1.1
*istB*		→	3.1	1.7	1.7	1.2
*aphA*	Aminoglycoside 3′-phosphotransferase	→	1.1	1.1	0.9	1.2
*traA*		←	0.9	0.8	0.6	1.2
*traB*		←	1.0	1.1	0.9	1.4
*traC1*	DNA primase	←	1.0	1.0	1.3	1.5
*traC2*	DNA primase	←	0.8	1.1	1.3	1.4
*traD*		←	1.1	1.1	1.6	1.2
*traE*	Putative helicase	←	1.8	1.5	1.4	1.5
*traF*	P-type propilin processing	←	2.7	2.0	2.6	1.3
*traG*	Coupling protein	←	**4.2**	2.8	2.6	1.4
*traI*	Relaxase	←	**22.1**	**6.5**	**14**.**6**	1.5
*traH*	Relaxosome auxiliary protein	←	**21.1**	**6.3**	**15**.**4**	1.2
*traJ*	Relaxosome auxiliary protein	←	**15.1**	2.6	**6**.**0**	1.1

**FIGURE 2:**
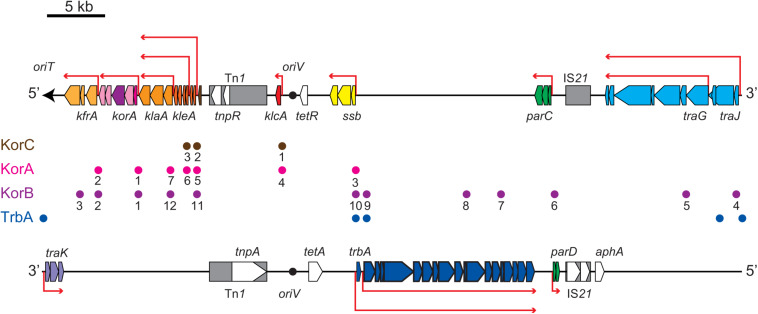
Scheme of RP4 genes encoded on the transfer strand (upper) and the complementary strand (lower). The origin of transfer (*oriT*) and its direction is indicated by the black arrow. The origin of replication (*oriV*) is indicated by the black circle. The location and length of representative RP4 genes are indicated by pentagons in the direction of transcription on their respective template strand (antisense strand). Transposon and insertion sequence are shown in gray boxes. Red arrows indicate representative polycistronic transcription units. Circles between the transfer and complementary strands indicate the operator sequences of the global regulators, KorA (magenta), KorB (purple), KorC (orange), and TrbA (blue). The KorA, KorB and KorC operators (O_A_, O_B_, and O_C_) are numbered as in Pansegrau et al. (1994a).

*The RP4 gene name and direction of transcription relative to the direction of transfer are indicated on the left columns. Combinations of donor and recipient strains are indicated in the upper rows. ^*a*^The direction of transcription corresponds to that shown in Figure 2. ←, mRNA is transcribed from the transfer strand as template; →, mRNA is transcribed from the complementary strand as template. Fold changes >4.0 are shown in bold.*

Our microarray analysis clearly showed strong zygotic induction specifically on the leading region of transfer strand, namely, *kfrABC*, *korA-incC-korBFG*, *klaABC*, and *kleABCDEF* operons, which are involved in stable inheritance of the plasmid (Wilson et al., 1997; Adamczyk et al., 2006). Since the leading region enters into the recipient cells in the early stage (Figure 2), the zygotic induction of these operons might be advantageous to the plasmid establishment in the new recipient cells. Among the induced operons, the *kor* operon encodes the KorA and KorB transcriptional regulators, which bind to 7 and 12 operator sequences on RP4, respectively (Kornacki et al., 1993; Jagura-Burdzy and Thomas, 1994, 1995; Jagura-Burdzy et al., 1999b; Kostelidou et al., 1999; Kostelidou and Thomas, 2000, 2002; Bingle et al., 2005; Chiu et al., 2008). Between *korA* and *korB* genes, the operon also encodes the IncC plasmid partitioning ATPase, which interacts with KorB (Motallebi-Veshareh et al., 1990; Jagura-Burdzy et al., 1999a; Rosche et al., 2000). Zygotic induction of KorA and KorB repressors suggests that these global regulators together allow only a temporal expression of their target genes on RP4 in the early stage of conjugative transfer. Interestingly, chromatin immunoprecipitation analysis has revealed that KorB transcriptional regulator from RP4 binds on an operator sequence found in *P*. *putida* KT2440 chromosome (Chiu and Thomas, 2004). However, we found no chromosomal genes that exhibit significant changes in common in our microarray data (data not shown).

**FIGURE 3:**
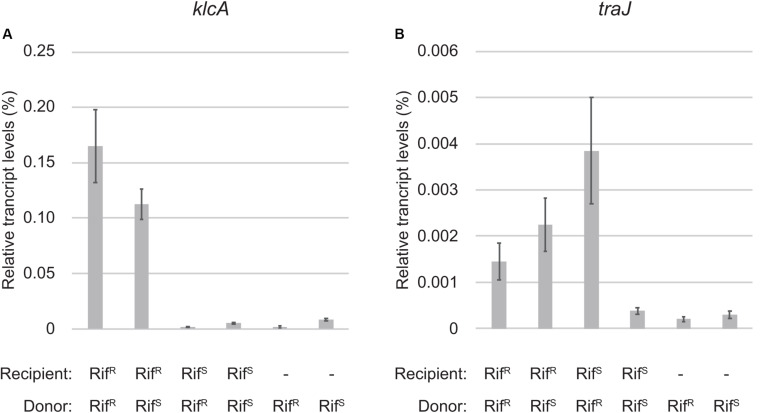
Quantitative RT-PCR analysis of zygotic induction. The transcript levels of *klcA* **(A)** and *traJ* **(B)** mRNAs relative to 16S rRNA are shown. The combinations of Rif^R^ and Rif^S^ strains for donor and recipient of RP4 are indicated below. The two bars on the right are control samples of donor cells only.

The most strongly induced gene was *klcA*, which is encoded ∼20 kb away from *oriT* on the transfer strand. Under steady-state conditions, *klcA* was transcribed at one of the lowest basal levels among the RP4 genes (Supplementary Table S1), which is attributable to the strong repression by KorA and KorC (Figurski et al., 1982; Thomas et al., 1988; Kornacki et al., 1993). The KlcA protein has recently been shown to exhibit an antirestriction activity (Goryanin et al., 2018). RT-qPCR analysis confirmed that the *klcA* transcript is induced ∼120-fold in the transconjugant cells (Figure 3A). The surge of KlcA expression might be beneficial to prevent the cleavage of double-stranded plasmid DNA by restriction enzymes in a new host. It is important to note that not all the genes on the transfer strand were induced during the conjugation, e.g., *bla* and *korC*. This result is in line with the fact that Tn*1* transposon insertion interrupts the transcription of *klcA* operon and the constitutive *bla* mRNA reads through the downstream *korC* gene in IncP-1α plasmids (Kornacki et al., 1990). The constitutively expressed KorC might be responsible for the very low basal level of *klcA* transcript.

We also observed the zygotic induction of *trfA* operon, which is composed of *ssb*, *trfA*, and upf16.5 (Table 1). The *trfAp* promoter is strongly repressed by KorA and KorB proteins cooperatively by binding at O_B_10 (Jagura-Burdzy and Thomas, 1994, 1997; Jagura-Burdzy et al., 1999b). The O_B_10 site exhibits the highest affinity for KorB (Kostelidou and Thomas, 2000). The *trfA* gene expresses two isoforms both of which bind on the *oriV* region to initiate vegetative plasmid replication (Pansegrau et al., 1994a; Thorsted et al., 1996). The first gene *ssb* encodes the single-stranded DNA (ssDNA) binding protein, which is probably involved in vegetative replication of RP4 (Jovanovic et al., 1992) or might play a role in conjugative transfer by protecting the transferred ssDNA. The zygotic induction of SSB encoded in the leading region has also been observed in F and ColIb-P9 conjugative plasmids (Bagdasarian et al., 1992; Jones et al., 1992; Althorpe et al., 1999) through stimulation of single-stranded promoters (Masai and Arai, 1997; Bates et al., 1999; Nasim et al., 2004). However, we have not identified such promoters for the zygotically induced RP4 genes so far.

On the complementary strand, we detected a modest induction of the *trb* operon encoding the components for Mpf/T4SS to transport the plasmid ssDNA linked with the relaxosome protein complex. The *trb* operon has two promoters, *trbAp* and *trbBp*, and can be transcribed only after the template strand is replicated in the new transconjugant cells. The relatively strong *trbBp* responsible for the transcription of *trb* operon is cooperatively repressed by TrbA and KorB, i.e., KorB alone represses *trbBp* only weakly by binding at O_B_9 (Zatyka et al., 1997, 2001; Bingle et al., 2003, 2005). *trbAp* is located face-to-face with the strong *trfAp* promoter, which inhibits the activity of *trbAp* via elongating transcription complexes in the opposite direction. *trbAp* can be activated through inhibition of counteracting *trfAp* by KorA and KorB proteins (Jagura-Burdzy and Thomas, 1994, 1997; Jagura-Burdzy et al., 1999b), implying that transcription of *trbA* is allowed after the repression of *trfAp* is completed. Therefore, we could only detect zygotic induction of transcripts originated from *trbBp*, and transcription from the upstream promoter *trbAp* was not induced in 10 min. Moreover, the induction rate went down below the threshold as the transcription proceeded into downstream genes (Table 1). We expect to detect the late induction of *trb* operon at higher levels by increasing the duration of filter mating.

Both the divergently transcribed *parCBA* and *parDE* operons encoding the multimer resolution system and the post-segregational killing system, respectively, contribute to the stable inheritance of RP4 plasmid (Gerlitz et al., 1990; Roberts and Helinski, 1992; Eberl et al., 1994; Jovanovic et al., 1994; Roberts et al., 1994; Sia et al., 1995). Autogenous regulation of the divergent promoters by ParA and ParD (Davis et al., 1992; Eberl et al., 1992) accounts for the modest induction of this locus in the transconjugant cells (Table 1), while this locus contains a low-affinity binding site for KorB(O_B_6) whose contribution to transcription regulation remains unknown (Kostelidou and Thomas, 2000).

### Zygotic Induction in Donor Cells

The trailing region, which enters the recipient in the end of plasmid transfer, harbors the *traJIHGFEDCBA* operon encoding the components of relaxosome and other Dtr proteins. Upon binding of auxiliary proteins TraJ and TraK at *oriT*, TraI relaxase is recruited to *oriT* to form the relaxosome (Fürste et al., 1989). Binding of TraJ protein at the 19-bp inverted repeat interspaced by 8 bp to the *nic* site is required for the strand-specific cleavage by TraI relaxase (Ziegelin et al., 1989; Pansegrau et al., 1990b). The interaction of TraJ and TraI at *oriT* is stabilized by the acidic protein TraH, which is encoded in a different reading frame within the *traI* gene, to form the relaxosome nucleoprotein structure (Pansegrau et al., 1990a). TraI cleaves the *nic* site in a site- and strand-specific manner and covalently binds with the 5′ end of the transfer strand at its 22nd tyrosine residue (Y22) in the catalytic center. The relaxosome is recruited to T4SS by the coupling protein TraG (Balzer et al., 1994; Schröder et al., 2002; Schröder and Lanka, 2003).

The *tra* operon is transcribed from the upstream *traJp* and downstream *traGp* promoters (Figure 2). Importantly, the relaxase gene *traI* is solely transcribed from *traJp*, while the coupling protein gene *traG* is transcribed in two different mRNAs. Microarray analysis revealed a strong induction of *traJ*, *traI*, and *traH* genes in the mating between the Rif^R^ recipient and Rif^R^ donor cells (Table 1). Unexpectedly, in the mating between the Rif^S^ recipient and Rif^R^ donor, where the conjugative transfer reaction stops after the first reaction (Figure 1C), we observed specific induction of *traJ*, *traH*, and *traI* but not the other RP4 genes. Because no transcription can be initiated in Rif^S^ transconjugant cells in the presence of Rif, this result indicates that *traJp* is activated in donor cells during conjugative transfer. Indeed, we found no plasmid genes that showed significant expression changes in the combination of Rif^S^ donor and Rif^S^ recipient (the rightmost column of Table 1). We verified by RT-qPCR analysis that the *traJ* transcript was strongly induced in the conjugating Rif^R^ donor cells (Figure 3B). The transcriptional induction of *traJ* was also observed in the mixture of the Rif^S^ donor and Rif^R^ recipient cells, which might reflect the expression in the new Rif^R^ transconjugant cells.

**FIGURE 4:**
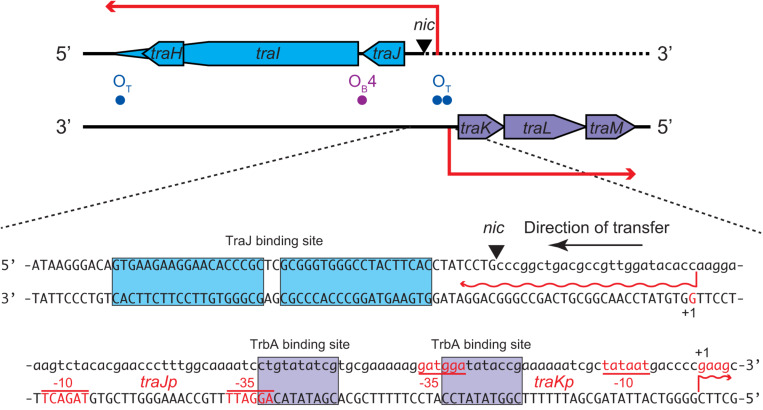
Nucleotide sequence of the *oriT* region of RP4 plasmid. The *nic* site cleaved by TraI is indicated by the triangle. The nucleotides of the transfer strand which are transported into recipient first are written in lowercase. The transcription start sites and promoter sequences as previously reported (Zatyka et al., 1994) are indicated as +1, –10, and –35, respectively, on each non-template strand. The TraJ and TrbA binding sites are boxed. The binding site of TraK is located downstream of *traK* transcription start site (not shown).

The *oriT* of RP4 contains divergent promoters, *traJp* and *traKp*, which are regulated by a complex of multiple regulatory proteins (Figure 4). Binding of TraJ protein at the 19-bp inverted repeat causes autorepression of *traJp* (Zatyka et al., 1994). TraK protein binds the intrinsically curved ∼200-bp *oriT* region downstream of *traKp* (Ziegelin et al., 1992) and represses both *traJ* and *traK* (Zatyka et al., 1994). In addition, TrbA binds at two sites overlapping the -35 boxes of *traJp* and *traKp* (Bingle et al., 2003). *traGp* is repressed by KorB through binding its operator sequence (O_B_4) in the *traJ-traI* intergenic region without affecting the activity of *traJp* (Bingle et al., 2005). Interestingly, the transcription initiation site of *traJ* has been reported to locate at the G nucleotide 24-nt upstream of the *nic* site (Greener et al., 1992; Zatyka et al., 1994). Given that the first 24 nt of 5′ untranslated region of *traJ* is encoded on the transfer strand (Figure 4), it is impossible to transcribe *traJ* in the absence of the leader region of the transfer strand. Our 5′RACE analysis verified that the *traJ* transcripts were homogeneously accumulated in the donor cells during conjugative transfer and started from the same nucleotide as previously reported (data not shown). Therefore, it is most likely that the *tra* operon is induced in the donor cell immediately after regeneration of the *traJ* promoter from the 3′ end of *oriT*, which in itself dissociates the autogenous repressor proteins from *oriT*. Since TraI associates with both ends of *oriT* to circularize the plasmid under steady-state conditions, we envisage that the *oriT* region becomes accessible to replication and transcription machineries after the relaxosome complex is transferred into the recipient cell.

### A Model of Continuous Plasmid Transfer by Induction of Relaxase Operon

Conjugative transfer accompanies rolling-circle replication (RCR) to generate two identical copies of plasmid DNA in recipient and donor cells (Willetts and Wilkins, 1984; Lanka and Wilkins, 1995). As the transfer strand is initially cleaved and bound with a relaxase, both replacement strand and complementary strand are synthesized in the cells, which are directly connected but segregated by the membranes. In the recipient cell, the 5′ and 3′ ends of the transfer strand are ligated and recircularized by the relaxase that is transported together to create a unit-length copy of plasmid DNA, and the complementary strand is replicated from RNA primers, which are presumably generated by a plasmid-encoded DNA primase (Rees and Wilkins, 1990). In the donor cell, the 3′ end of transfer strand acts as a primer for replacement strand synthesis by a DNA polymerase III (Pansegrau et al., 1990b). However, it remains paradoxical in which cell the relaxase executes the second cleavage reaction, which is a prerequisite to generate a unit-length plasmid copy and terminate RCR (Chandler et al., 2013). In the F plasmid transfer system, it has been proposed that the second cleavage reaction is likely to occur in the donor cell rather than in the recipient cell (Dostál et al., 2011). In contrast, the TrwC relaxase of R388 plasmid system has been shown to be transported into the recipient cell and then recircularize the transferred DNA (Draper et al., 2005). Although both TraI_F_ and TrwC relaxases contain helicase domains and are categorized into the same MOB_F_ family (Garcillán-Barcia et al., 2009), this inconsistency between the two systems might be attributable to the number of active tyrosine residues required for the cleavage reaction by the relaxase. TrwC employs Y18 for the initial cleavage and Y26 for the second cleavage in the same molecule and, therefore, is capable of the transfer termination in the recipient cell (Gonzalez-Perez et al., 2007). However, among two pairs of tyrosines (Y16, Y17, Y23, and Y24) in its transesterase domain of TraI_F_ relaxase, Y16 is the only residue critical for conjugative transfer (Dostál et al., 2011). Once covalently attached with the 5′ end of ssDNA, the single active tyrosine residue is unable to catalyze the second cleavage reaction, raising the possibility that a second tyrosine residue is provided by another relaxase protomer or is substituted by an alternative nucleophile such as water (Chandler et al., 2013). Recently, it has been solved that the full-length TraI_F_ forms a dimer to bind both 5′ and 3′ ends of *oriT* simultaneously by adopting closed and open conformations, respectively (Ilangovan et al., 2017). This implies that one molecule of TraI_F_ is left behind in the donor cell to cleave the newly synthesized *oriT* and produce the unit-length plasmid DNA.

**FIGURE 5:**
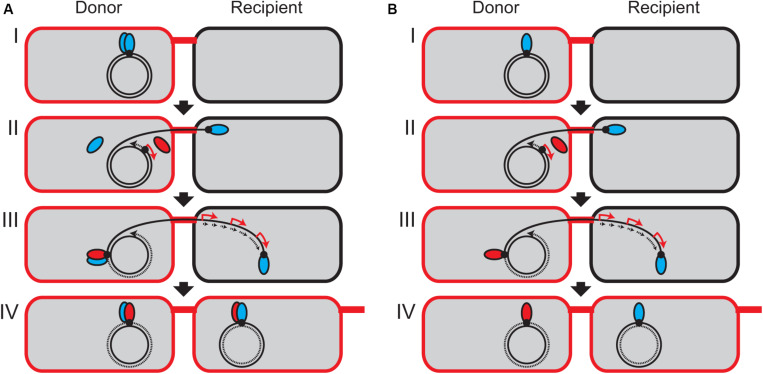
Model of continuous plasmid transfer by induction of relaxase in the donor cell. It has not been experimentally verified whether TraI forms a dimer **(A)** or a monomer **(B)** *in vivo*. **(I)** At the *oriT* site (black dot), the TraI relaxase (blue ellipsoid) is covalently bound to the 5′ end of tranfer strand. **(II)** One relaxase starts to be transported from the donor into the recipient. The 3′ end of transfer strand serves as a primer to initiate the replication of replacement strand (dotted arrow). Since the *traJ* promoter region is the first to be replicated, the transcription of *traJIH* operon is temporally stimulated (red arrow) to express the second TraI relaxase (red ellipsoid). It is unknown whether only one TraI molecule has been transported into the recipient cell through the T4SS machinery. **(III)** In the donor cell, the induced relaxase or the remaining free monomer binds at the *oriT* site to reconstitute a new relaxosome complex and generates the unit-length ssDNA to terminate the plasmid transfer. In the recipient, the complementary strand is replicated (dotted arrows) and the transcription of plasmid genes are induced (red arrow). **(IV)** The rolling-circle replication is accomplished in the donor cell, and the transferred strand is recircularized in the recipient cell to be established as the new transconjugant cell.

In the case of RP4 plasmid transfer system, the TraI relaxase contains only a single tyrosine residue Y22, which catalyzes the cleavage at *nic* and covalently binds with the 5′ end of the nicked DNA strand (Pansegrau et al., 1993, 1994b). It has been demonstrated by *in vitro* assay using a magnetic bead technique that TraI existing as a monomer in solution is unable to conduct the second cleavage reaction (Pansegrau and Lanka, 1996). Unlike the MOB_F_ family relaxases, the RP4 TraI does not have a helicase activity, and the conformation of TraI bound with *oriT* ssDNA remains ambiguous. Here, we propose a model that after the relaxase linked with transfer strand is transported into the recipient cell, the expression of the second copy of relaxase is induced at the transcriptional level in the conjugating donor cell to replenish the first relaxase (Figure 5). If the relaxase exists as a dimer *in vivo*, either the induced relaxase or the remaining monomer is responsible for the second cleavage in the donor cell (Figure 5A). Even if the relaxase exists as a monomer, a small population of TraI molecules could provide a second tyrosine residue for the cleavage of the other plasmid copies (Figure 5B). However, it should be noted that the copy number of RP4 plasmid is estimated at less than three copies in *Pseudomonas* spp. (Itoh et al., 1984), and the translation of TraI is limiting (Pansegrau et al., 1990a). Further study is required to determine the exact copy number of TraI molecules in the RP4 donor strain and investigate whether or not the induced relaxase is responsible for the second cleavage reaction.

This model is not contradictory to the previous results that *de novo* expression in the donor cell is dispensable for initiation of conjugative transfer (Wilkins and Hollom, 1974; Kingsman and Willetts, 1978). Pretreatment of donor cells with Rif did not prevent the initiation and termination of plasmid transfer given the presence of free TraI molecules. We also note that this model is not the case for other plasmid systems such as R388 (Draper et al., 2005). Nonetheless, the feature of *oriT* region with a pair of divergent promoters can often be seen in diverse groups of conjugative plasmids (Lanka and Wilkins, 1995; Francia et al., 2004). It is tempting to speculate that zygotic induction of relaxase in donor cells facilitates conjugative transfer in general.

## Conclusion

This study revisited the phenomenon known as zygotic induction during conjugative transfer of plasmid RP4. By transcriptomic analysis, we have detected strong induction of several operons in the transconjugant cells. This is attributable to derepression of transcription by plasmid-encoded repressor proteins. We have also revealed that the conjugating donor cells induce the transcription of *traJIH* operon, which is initiated from the *oriT*-proximal promoter. This mechanism shed light on the long-standing question over the requirement of the second relaxase molecule for the termination of conjugative transfer. Since this study has only detected the transcripts in the mixtures of recipient and donor cells, further study is required to visualize the relaxase molecules associated with the plasmid transfer strand *in vivo* at a single-cell level. Overall, this study provides new insights into the differential regulation of plasmid gene expression in donor and recipient cells during conjugative transfer. Our methodology is applicable for many conjugative plasmids to analyze their dynamic expression in minute detail using current RNA-seq technologies.

## Data Availability Statement

The data are deposited in NCBI’s Gene Expression Omnibus (GEO, http://www.ncbi.nlm.nih.gov/geo/) and are accessible through GEO Series accession number GSE146879.

## Author Contributions

MM conceived the study, designed and performed the experiments, and wrote the manuscript. YO, YN, and MT contributed to discussion, editing, and revision of the manuscript.

## Conflict of Interest

The authors declare that the research was conducted in the absence of any commercial or financial relationships that could be construed as a potential conflict of interest.
